# Novel Target Study to Cure Cardiovascular Disease regarding Proprotein Converse Subtilisin/Kexin Type 9

**DOI:** 10.1155/2022/9039377

**Published:** 2022-10-11

**Authors:** Yingjing Zhao, Weihang Li, Weiye Li, Hong Tao, Yuting Li, Bo Wu, Xinhui Wang, Huasong Zhou, Bo Gao

**Affiliations:** ^1^Department of Critical Care Medicine, Nanjing First Hospital, Nanjing Medical University, Nanjing, Jiangsu, China 210006; ^2^Department of Orthopedic Surgery, Xijing Hospital, Air Force Medical University, Xi'an, China; ^3^Clinical Medical School, China-Japan Union Hospital of Jilin University, 126 Xian Street Changchun 130033, Changchun, Jilin, China; ^4^Department of Orthopaedics, The First Hospital of Jilin University, Changchun, Jilin, China; ^5^Department of Oncology, First People's Hospital of Xinxiang & The Fifth Affiliated Hospital of Xinxiang Medical College, Street Yiheng, 63 Xinxiang, China; ^6^Department of General Surgery, Xi'an Hospital of Traditional Chinese Medicine, Xi'an, Shaanxi, China

## Abstract

**Objective:**

This study is aimed at screening the potential ideal lead compounds from natural drug library (ZINC database), which had potential inhibition effects against proprotein converse subtilisin/kexin type 9 (PCSK9), and contributing to enrich the practical basis of PCSK9 inhibitor screening.

**Methods:**

A series of computer-aided virtual screening techniques were used to identify potential inhibitors of PCSK9. Structure-based virtual screening by LibDock was carried out to calculate the LibDock scores, followed by ADME (absorption, distribution, metabolism, and excretion) and toxicity predictions. Molecule docking was next employed to demonstrate the binding affinity and mechanism between the candidate ligands and PCSK9 macromolecule. Finally, molecular dynamics simulation was performed to evaluate the stability of ligand-PCSK9 complex under natural circumstance.

**Results:**

Two novel natural compounds ZINC000004099069 and ZINC000014952116 from the ZINC database were found to bind with PCSK9 with a higher binging affinity together with more favorable interaction energy. Also, they were predicted to be non-CYP2D6 inhibitors, together with low rodent carcinogenicity and AMES mutagenicity as well as hepatotoxicity. Molecular dynamics simulation analysis demonstrated that these two complex ZINC000004099069- and ZINC000014952116-PCSK9 had more favorable potential energy compared to the reference ligand, which could exist stably whether in vivo or in vitro.

**Conclusion:**

This study elucidated that ZINC000004099069 and ZINC000014952116 were finally screened as safe and potential drug candidates, which may have great significance in the development of PCSK9 inhibitor development.

## 1. Introduction

Atherosclerosis is a chronic inflammatory disease with large/medium size of arteries, characterized by the detention of modified lipoproteins in the arterial wall, which could lead to ischemic heart disease and strokes as well as peripheral vascular disorders, collectively named as cardiovascular disease (CVD) [1, 2]. Lipoprotein is involved in the formation of atherosclerosis and plays a pivotal role in plaque rupture, which is a common pathophysiological indicator of acute ischemic syndrome [3], among which low-density lipoprotein cholesterol (LDLc) is a type of the lipoprotein; lowering LDLc could decrease the risk of CVD [4, 5], such as stroke, which is the fifth leading cause of death in 2017 in the United States [6]; atherosclerosis; and myocardial infarction [7, 8]. Human PCSK9 gene, namely, proprotein converse subtilisin/kexin type 9, is mainly synthesized and secreted by the liver and is one of the key modulators of LDLc; besides, PCSK9 is also found to be closely connected with series of pathophysiological processes, like brain development, platelet activation, intestinal physiology, pancreas, and adipose tissue as well as neoplasms [2], suggesting that PCSK9 is the key regulatory target among different diseases. Existing genetic and interventional researches have fully reported that reducing the levels of PCSK9 corresponds to CVD benefits [2].

LDLc is eliminated through LDL-R recycling [9], while this process could be altered negatively by PCSK9 through degrading LDL-R. When PCSK9 capture the LDL-R/LDLc complex, it could further combine with the complex closely and then form a novel complex PCSK9/LDL-R/LDLc, which is then internalized through the cell membrane and sent to the lysosome for degradation, resulting in the degradation of LDL-R, thus preventing LDL-R from recycling to the cell membrane [10, 11]. Consequently, decreasing the degradation of LDL-R by PCSK9 inhibitor could help LDLc cleaning and eventually reduce the risk of atherosclerosis [12, 13]. These findings implied that PCSK9 inhibition could be a potential and effective therapeutic target to cure or prevent CVD in individuals with high levels of PCSK9.

Recent researches showed that the current primary pharmacological inhibitors of PCSK9 such as evolocumab and alirocumab were monoclonal antibodies, which were potent in the LDL-lowering process, together with good tolerance by patients [14]. However, monoclonal antibodies still have some disadvantages like the high cost, injection site adverse reaction, and no oral administration approach. The expensive as well as inconvenient situation makes it hard for patients to receive this therapeutic approach widely. Current methods in lowering LDLc level include inhibiting the function and influencing the synthesis as well as processing of PCSK9 [15], interfering the PCSK9/LDLR protein-protein interaction, and silencing PCSK9 gene expression by genetic alteration such as siRNA [16]. However, most of these approaches did not emerge promising effects because of limitations, such as potential off-target mutagenesis for disrupting PCSK9 by gene genome editing and instability in plasma parenteral administration for small peptide [13, 16].

Nature products and their derivatives play a crucial role in today's pharmacologic market, small molecules are pivotal aspect if not the first means to tackle an emergent or unpredictable diseases, and they have still made a great contribution to medication design and improvement [2, 17, 18]. Novel nature inhibitors targeting PCSK9 may benefit from these aspects: newly aromatic compounds from the fruiting body of Sparassis crispa, berberine, and inclisiran are reported to be potent PCSK9 inhibitors, which can influence PCSK9 mRNA expression [15, 19–21]. Imidazole-based minimalist peptidomimetic and truncated LDL-R EGF-A-domain peptides can disrupt the PCSK9/LDLR protein-protein interaction [22]. However, a suitable novel nature inhibitor targeting PCSK9 was hard to discover without comprehensive and professional evaluation, not to mention further in vivo studies. Currently, only small amount of PCSK9 inhibitor researches were found to be relatively mature, such as polydatin and tetrahydroxydiphenylethylene-2-O-glucoside [23, 24]. Therefore, there still needs more study to screen the potential PCSK9 inhibitors as well as analyze possible mechanism of the interactions.

Structural biology study is an effective way on the basis of high-throughput techniques, to screen nature compounds targeting specialized protein molecules from huge of ligands, which avoid the large amount of manpower, materials, and financial resources required for traditional drug screening (manual drug addition experiments). Current computational simulation study on PCSK9 inhibitors include Exploring Key Orientations (EKO) and computational GOLD algorithm analysis [25, 26]. This study performed different chemical molecule database and computational methods to discover potential candidate compounds, aiming to screen potential lead compounds with well binding affinity and effective functions as well as existence of stability under natural environment. A set of virtual screening, molecular docking, toxicity prediction, and ADME model was fully performed to screen the promising compounds targeting PCSK9; then, ligand binding analysis and molecular dynamics simulation were used to understand the mechanism further. A reported inhibitor of PCSK9 was chosen as reference to make comprehensive evaluation for novel ligands and existing inhibitors of PCSK9 [27].

## 2. Results

### 2.1. High-Throughput Screening of Natural Product Database against PCSK9

**Figure 1:**
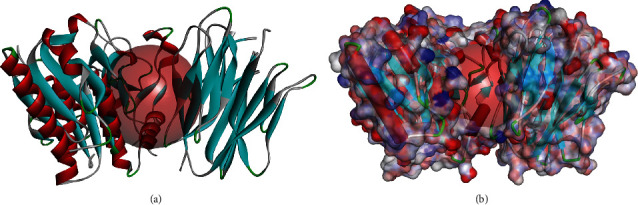
Molecular structure of proprotein converse subtilisin/kexin type 9 (PCSK9): (a) initial molecular structure; (b) surface of binding area was added, blue indicated positive charge, and red indicated negative charge.

**Figure 2:**
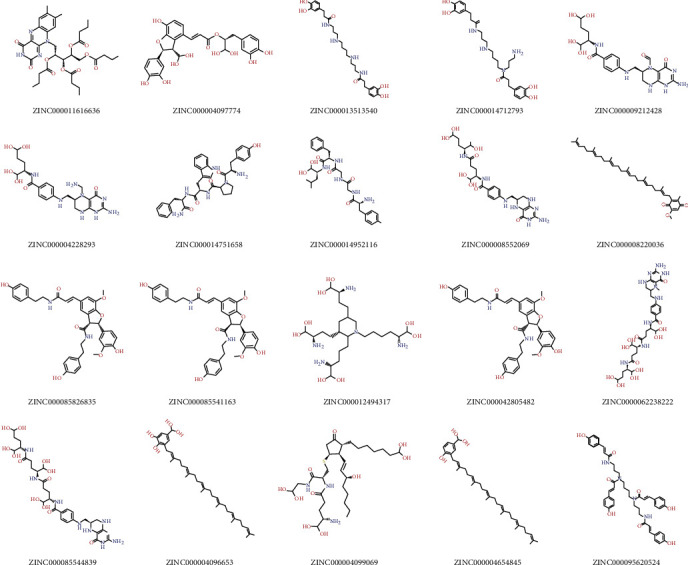
The chemical structures of the top 20 compounds.

Chemical structure of PCSK9 is displayed in Figures 1(a) and 1(b), the existed ligand-binding pocket was an essential active regulatory site of PCSK9, and small ligands binding to this region could change the conformation of the protein and thus inactivate the activity of PCSK9, so the initial ligand from PCSK9 complex was extracted and the region was set as the binding sphere. Totally, 17776 purchasable-natural-named products were obtained from ZINC repository for research. With high-throughput screening, each of these ligands was put into the binding sphere to bind with the protein, and finally, 13430 compounds were found to bind eligibly with PCSK9 through the screening algorithm; among those, 2081 ligands had higher LibDock scores than the reference ligand (LibDock score: 124.227). The top 20 compounds with the highest LibDock scores are listed in Table 1, together with these chemical structures of these potential lead compounds (Figure 2).

### 2.2. Pharmacological Properties and Toxicity Prediction

Pharmacological properties of these ligands were fully evaluated through ADME (absorption, distribution, metabolism, and excretion) algorithm; these indicators include solubility level, brain/blood barrier (BBB) level, cytochrome P450 2D6 (CYP2D6) prediction, hepatotoxicity, absorption level, and toxicity properties. As shown in Table 2, all compounds could pass through the BBB indicated by BBB level (score: 4); solubility level showed that all compounds were soluble in water except ZINC000008220036; three compounds were predicted to be inhibitors of CYP2D6, which had an important role in drug metabolism; and seven compounds were predicted to be hepatotoxic; seventeen compounds were found to have a higher absorption level compared to the rest of three compounds; as for the reference ligand, it was predicted to be toxic to the liver and non-CYP2D6 inhibitors.

Toxicity of candidate drugs also needs to be considered when screening potential compounds, through TOPKAT module (Table 3); indicators like AMES mutagenicity (AMES), developmental toxicity potential (DTP), and rodent carcinogenicity (based on the US. National Toxicology Program dataset) were included to ensure the safety of these potential drugs. Results revealed almost all drugs had developmental toxicity potential except ZINC000008220036; the reference ligand had high probability of DTP and AMES mutagenicity.

### 2.3. Ligand Binding Analysis

**Figure 3:**
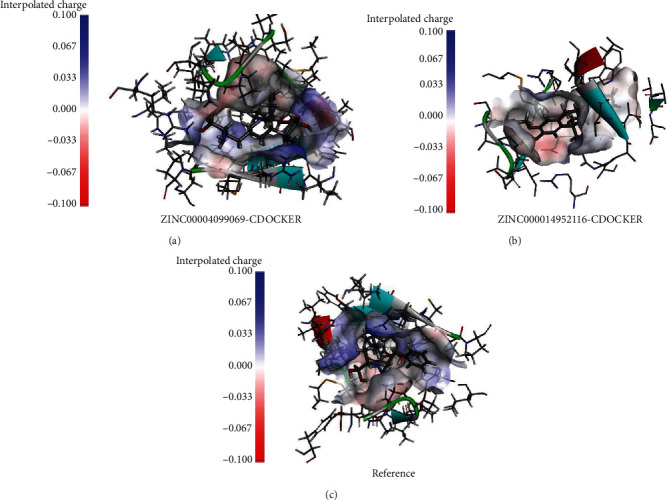
Schematic diagram of PCSK9 and the interaction of ZINC000004099069 (a), ZINC000014952116 (b), and reference (c) with PCSK9.

**Figure 4:**
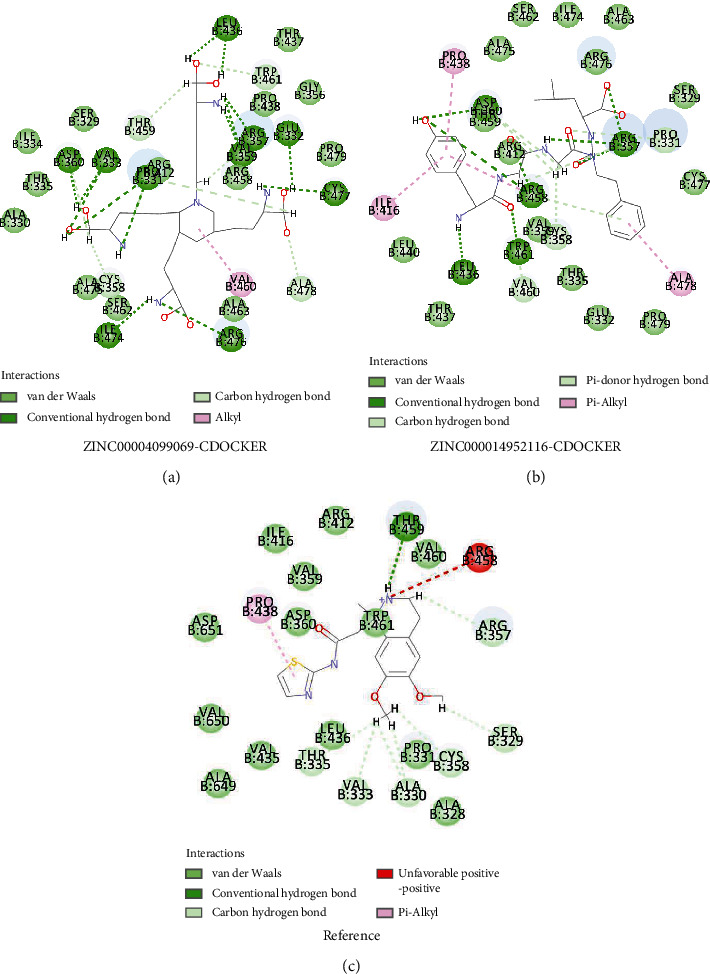
The intermolecular interaction of the predicted binding modes of ZINC00004099069 (a), ZINC000014952116 (b), and reference (c) with PCSK9.

To further understand the mechanism of the interaction between these candidate compounds with PCSK9, CDOCKER modules were conducted to make a precise docking algorithm, which could generate more accurate chemical bonds between ligand and protein and caused more running time. After the reference ligand redocking into the binding region of PCSK9, RMSD between initial ligand and docked posture was calculated as 0.92 Å, proving that the docking program applied in this study was highly reliable. Then, CDOCKER interaction energy was calculated to verify the binding affinity of ligands and PCSK9. CDOCKER module provided a 3D structure of the interaction between compounds and PCSK9, and CDOCKER interaction energy showed the affinity of potential compounds with PCSK9. The CDOCKER interaction energy of ZINC000004099069 with PCSK9 is -87.8609 Kcal/mol, lower than the CDOCKER interaction energy of ZINC000014952116 with PCSK9, -65.9632 Kcal/mol, which meant that the former complex could bind with PCSK9 better (Table 4). The hydrogen bonds and hydrophobic interactions formed by PCSK9, and these two compounds were visualized (Figures 3(a)–3(c)) and analyzed (Figures 4(a)–4(c)).  Table 5 displays that ZINC000004099069 formed 22 pairs of hydrogen bonds with PCSK9, and ZINC000014952116 formed 18 pairs of hydrogen bonds with PCSK9. The interaction between ZINC000004099069 and PCSK contains 1 hydrophobic interaction, and the interaction between PCSK and the other promising chemical molecular includes 4 hydrophobic interactions, as shown in Table 6.

### 2.4. Molecular Dynamics Simulation

**Figure 5:**
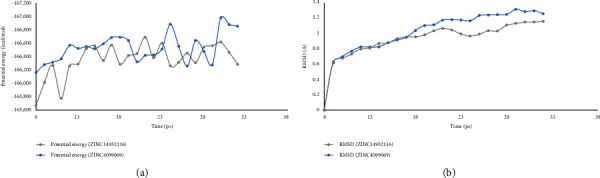
Results of MD simulation of ZINC000004099069 and ZINC000014952116.

Molecular dynamics simulation had been performed to further evaluate the stabilities of PCSK9-ligand complexes under natural situation. The initial conformation of these complexes was obtained from CDOCKER module, an accurate molecular docking program. RMSD curves as well as potential energy of ZINC000004099069- and ZINC000014952116-PCSK9 complexes are shown in Figure 5, the trajectory of each complex got stable gradually at about 20 ps, and then, they both went to equilibrium. It was convincing that through these MD simulation procedures, hydrogen bonds and  *π*-related chemical bonds between ligands and PCSK9 could enhance the interactions within the complex and thereby contributed the stabilities of complexes. MD simulation results suggested both of the two compounds could interact stably with PCSK9, and ligand-PCSK9 complexes could exist steadily under natural situation. Considering all results above, ZINC000004099069 and ZINC000014952116 were finally selected as potential lead compounds with less rodent carcinogenicity, hepatotoxicity, AMES mutagenicity, and good solubility and intestinal absorption level; they were not toxic to the liver and did not behave as CYP2D6 inhibitors. Additionally, in terms of molecular dynamics simulation, the formed complex could also behave stable performance under natural situation.

### 2.5. Validation of the Effects of the Candidate Compounds

**Figure 6:**
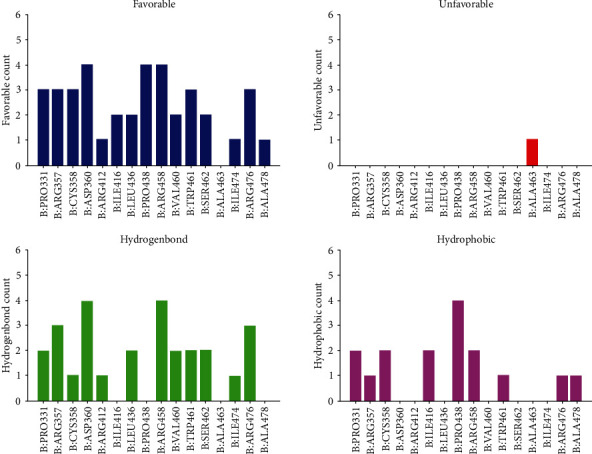
Schematic intermolecular interactions of the candidate compounds with PCSK9.

This study recruited the top 20 compounds with the highest LibDock scores conducted by high-throughput screening and then put them into a more reliable algorithm “analyze ligand poses,”, to test the binding affinity of ZINC000004099069 and ZINC000014952116 compounds. After calculating the residues and PCSK9 receptor, there displayed favorable and unfavorable count and hydrophobic count as well as hydrogen count, respectively. As shown in Figure 6, results validated that ZINC000004099069 and ZINC000014952116 compounds had the most active residues and the least unfavorable residues relatively, proving the reliability of these two candidate compounds.

### 2.6. Possible Pharmacophore Modification of the Lead Compounds

**Figure 7:**
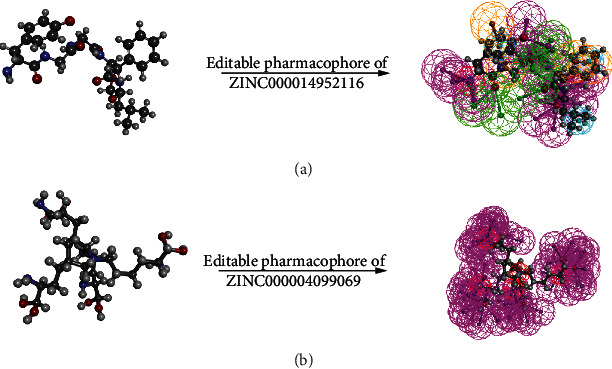
Pharmacophore predictions of ZINC000014952116 (a) and ZINC000004099069 (b) using 3D-QSAR. Green represents hydrogen acceptor, blue represents hydrophobic center, purple represents hydrogen donor, and orange represents aromatic ring.

After screening the potential lead compounds of PCSK9, this study further analyzed the editable skeleton of these two compounds through pharmacophore properties, to observe the possible modification site. As shown in Figures 7(a) and 7(b), results visualized that on the skeleton of these compounds, there were 106 editable features in ZINC000004099069 and 49 editable features in ZINC000014952116, among which, ZINC000004099069 had 40 hydrogen bond acceptors, 61 hydrogen bond donors, and 5 positive ionizable; ZINC000014952116 possessed 20 hydrogen bond acceptors, 19 hydrogen bond donors, 5 hydrophobic centers, and 1 positive ionizable as well as 4 ring aromatics, which could be further improved targeting these editable sites.

## 3. Discussion

PCSK9, mainly synthesized by the hepatocytes, is a pivotal regulator of LDL-R, which could reverse the clearance of LDLc [12, 13]. Many hypercholesterolemia patients possess a high level of PCSK9. Therefore, targeting the function of PCSK9 is a promising direction for lowering LDLc in order to further cure CVD. Up to now, there have been some targeted drugs of PCSK9, such as monoclonal antibodies (evolocumab and alirocumab) and nature inhibitors (polydatin and tetrahydroxydiphenylethylene-2-O-glucoside) [14, 23, 24]. The high cost of using monoclonal antibodies and the slow progression in putting natural inhibitors into clinical trial require more candidate natural drug research in this field. However, it is worth noting that most natural products generally lead to low aqueous solubility and poor stability as well as bioavailability like less cellular absorption and low intestinal absorption and also high molecular weight due to their physiochemical properties, which may block the development of natural drug screening [28, 29]; these aspects all prompt researchers to fully evaluate each compound when drug screening to discover the best effective candidate drugs. The high-throughput method used in this study could reduce the cost of medicine research and development, such as manpower and materials. Some studies have suggested a high priority of using structural biology method [2, 18, 30]. To the best knowledge, the researches using this analytical method to screen PCSK9 have not been reported so far; thus, this study could provide a novel insight for exploring targeted drug therapy of PCSK9 and contribute in this field.

In this study, a total of 13430 compounds of purchasable-natural-named products taken from the ZINC database were found to bind with PCSK9 eligibly. The top 20 of these compounds based on LibDock score were screened firstly and selected for further ADME and toxicity prediction. Through results, LibDock scores represented their binding affinity with protein PCSK9; compounds with higher LibDock score indicated a better energy optimization and more stable structure in its complex. This study chose the top 20 compounds with the best LibDock score for first screening, which were pooled for the following study.

ADME and toxicity predictions were employed for compounds to access their pharmacological properties. The results demonstrated that ZINC000004099069 and ZINC000014952116 were ideal compounds for high solubility, strong plasma protein binding affinity, and high absorption levels. At the same time, they were predicted to be non-CYP2D6 inhibitors, with low rodent carcinogenicity and AMES mutagenicity as well as hepatotoxicity. The high solubility and intestinal absorption level could promote the drug dissolution and absorption process, which could benefit from oral medication. Because of their noninhibition to CYP2D6, they were not easy to be accumulated in the liver. Additionally, these two compounds were assessed to have less AMES mutagenicity and rodent carcinogenicity, which presented their preferable safety characteristic. However, the two compounds still had a relatively high DTP, showing the possible risk of further usage; more refinements needed to be conducted in these skeletons to avoid further toxicity. Through trimming the molecular groups to overcome the deficiency of these compounds, they still had potential in PCSK9 inhibitor research and development.

Ligand binding analysis elucidated the mechanism of the interaction between ZINC000004099069 and ZINC000014952116 with PCSK9. The main interaction of ZINC000004099069 with PCSK9 was hydrogen bonds, while the interaction of ZINC000014952116 with PCSK9 was composed of hydrogen bonds and *π*-*π* bonds. From illustrations, we could observe that these two compounds formed many chemical bonds with the protein PCSK9, and more bonds suggested harder structure, which could not be easily broken by other factors. Additionally, the CDOCKER interaction energy illustrated that both ZINC000004099069 and ZINC000014952116 had a high affinity with PCSK9, of which the former is higher, which confirmed the two complexes' stability. For this reason, by binding with different ligands which had high affinity with PCSK9, the conformation of PCSK9 could be changed, resulting in inactivation of protein function and leading into the clearance of LDLc, and ultimately played a major role in the treatment of CVD disease.

Lastly, molecular dynamics simulation was used to assess the stability of these complexes under natural circumstances. The stable existence of a complex in the natural environment indicated that it could be metabolized in the body as a whole unit and the ligand could not be separated from the protein by some metabolism processes, inactivating the function of the ligand. RMSD curves as well as potential energy of these ligand-PCSK9 complexes suggested the stability alteration of this conformation; as the progress of molecular dynamics went on, such as heating and equilibrium procedure, some groups and chemical bonds from the complex might change slightly, like bond rotation or fold conformation change; these alterations might cause the change of potential energy and RMSD value; high fluctuations indicated the instability of the conformation. From the illustrations, we observed that RMSD curves as well as potential energy got stable gradually, elucidating that these two ligand-PCSK9 complexes might exist stably. Consequently, these two complexes could keep stable and have promotion under nature environment. Besides, the ligand pose analysis as well as pharmacophore modification analysis all validated our results that ZINC000004099069 and ZINC000014952116 were potential lead compounds with activities.

Currently, the medication screening and design by a computational-aided method are mainly focused on tumor field, while targeted drug on basic diseases had hardly been studied. This study screened two ideal lead compounds targeting PCSK9 from natural products, which had effective activity and may inactivate the function of PCSK9, and finally decreased the accumulation of LDLc in the body and took place in the treatment of CVD disease. In conclusion, from a series of computer-aided studies, ZINC000004099069 and ZINC000014952116, two compounds, were finally selected as safe and potential candidate drugs. Meanwhile, the information of other candidates provided in this study is listed in Tables 1–3, which can enrich the researches of PCSK9 and contribute a strong basis for PCSK9 inhibitor or other medication design and improvement. It is noteworthy that despite the disadvantages of some compounds analyzed from ADMET model in this study, it still provided a novel drug skeleton for medication design and refinement; different atoms or groups could be added or deleted from the skeleton to avoid the toxicity or other side effects. Thus, based on these two skeletons, more alterations and modifications on pharmacophore would be conducted to further improve the pesticide effects as well as reducing toxicity.

Although precise measurements and detailed designs had been conducted in this study, there were still some deficiencies due to the method of computational study. Animal model experiments were still needed to verify our research in the further. More indicators, like half-maximal inhibitory concentration and half-maximal effective concentration, need to be conducted to advance these two compounds to animals and eventually clinical application.

## 4. Methods and Materials

### 4.1. Docking Software and Ligand Library

Discovery Studio 4.5 software (BIOVIA, San Diego, California, US) applied computation-aided structural biologic analysis to protein and other compounds for docking, modeling, prediction, etc. Natural inhibitors of PCSK9 used in this study were selected by analyzing information from the ZINC database, a free repository containing numerous ligands for commercial utility. And CDOCKER is used to explore the interaction of compounds and proteins.

### 4.2. Structure-Based Virtual Screening Using LibDock

Ligand-binding pocket region of PCSK9 was selected as the binding site and was used to screen compounds that could potentially bind with and then inhibit PCSK9. LibDock was a program which was applied to screen small molecules virtually. Using polar probes, nonpolar probes, and a grid placed into the binding site, hotspots were calculated by LibDock for the protein. Ligands were arranged to form favorable interactions using these hotspots. For ligand minimization, the Smart Minimiser algorithm and CHARMm force field were used. All the ligands were ranked according to the ligand score after minimization. The 2.0 Å crystal structure of human PCSK9 was downloaded from the protein data bank (PDB ID: 6U3X) and imported to the working circumstance of LibDock. To prepare the protein, crystal water and other heteroatoms around the protein were removed, and then, protonation, ionization, hydrogen, and energy minimization were added. Using the binding site of prepared protein and ligands, the active site for docking was generated. Using LibDock, the prepared ligands at the defined active site was docked virtually. Based on the LibDock score, all docked poses were generated and the ligands were ranked.

### 4.3. ADME (Absorption, Distribution, Metabolism, and Excretion) Properties and Toxicity Prediction

The ADME module and TOPKAT module of Discovery Studio 4.5 were employed to calculate ADME and toxicity properties including absorption, distribution, metabolism, and excretion. The four ADME aspects included BBB (blood-brain barrier) level, CYP2D6 prediction, hepatotoxicity, absorption (intestinal absorption) level, solubility (defined at 25°C in water) level, and PPB (plasma protein binding) prediction. These characteristics were fully assessed when selecting appropriate drugs for PCSK9.

### 4.4. Molecule Docking Analysis

CDOCKER module in Discovery Studio, an implementation of a CHARMm-based docking tool, was employed for precise docking study between ligands and protein. During the docking process, the receptor held rigid, while the ligands were allowed to be flexible. The CHARMm energy and the interaction energy, which demonstrated the ligand binding affinity, were calculated for each complex pose. Because the fixed crystal water molecules might affect the combination between the receptor and ligand, they were generally removed in a rigid and semiflexible docking process. Additionally, the water molecules were removed, and then, hydrogen atoms were added to the protein for protein optimization.

### 4.5. Molecular Dynamics Simulation

After the most appropriate ligand-PCSK9 complex was obtained and selected from the above calculation, molecular dynamics simulation would be done for valuing their stabilities. They were put into an orthorhombic box and solvated using an explicit periodic boundary solvated water model. Then, solid chloride was placed in this box with an ionic strength of 0.145 to simulate the natural environment. The box was subjected to the CHARMm forced field and relaxed by energy minimization (1000 steps of the steepest descent and 1000 steps of the conjugated gradient), with the final RMS gradient of 0.08326. The system's temperature was slowly driven from 50 K to 300 K for 2 ns, and equilibration simulation was run for 1 ns. With a time step of 2 fs, the whole MD simulations were run for 40 ns. The results were saved every 2 fs. Using Discovery Studio 4.5 software (BIOVIA, San Diego, California, US), the structural properties, potential energy, and RMSD of MD trajectory were determined. The CHARMm force field was used for both receptors and ligands. The binding site sphere of PCSK9 was defined as the region that came with radius 5 Å from the geometric centroid of the ligands. During the docking process, the ligands could bind with the residues within the binding spheres. After the parts of identified site were determined, the parts would be prepared into the binding site of PCSK9. After the docking process, each ligand generated 10 docking poses. And the posture with the highest docking score and best affinity would be selected. Besides, the CDOCKER interaction energy of different poses was also taken into calculation.

### 4.6. Validation of the Effects of the Candidate Compounds

To further validate if the selected compounds were the effective drugs in this study, we next performed ligand pose analysis based on the top 20 compounds in high-throughput module and analyzed the residue interactions between each compound and PCSK9. The candidate compounds with the best binding affinity could be evaluated by counts with favorable and unfavorable residues with PCSK9.

### 4.7. Pharmacophore Predictions of the Lead Compounds

After comprehensive assessment of these two compounds, this study then analyzed their pharmacophore characteristics and editable site through 3D-QSAR pharmacophore algorithm, which could provide up to 255 fits per molecule to represent a small molecule; only fits with energy values within the threshold of 10 Kcal/mol were retained.

## 5. Conclusions

This study employed a series of high-throughput methods based on structural biology, like virtual screening, precisely molecular docking, ADME, and toxicity prediction, as well as molecular dynamics simulation to find novel natural inhibitors regarding protein PCSK9, in order to treat cardiovascular disease by inhibiting the function of PCSK9. Totally, two compounds, ZINC000004099069 and ZINC000014952116, were finally screened as safe drug candidates, which had great significance in contributing to the development of PCSK9 inhibitor.

## Figures and Tables

**Table 1:** Top 20 ranked compounds with higher LibDock scores than PCSK9.

Number	ZINC ID	Compounds	LibDock score
1	ZINC000062238222	5-Methyltetrahydropteroyltri-L-glutamate	239.613
2	ZINC000085544839	Thf-polyglutamate	238.17
3	ZINC000095620524	Tetra-*trans*-P-coumaroylspermine	226.594
4	ZINC000004099069	S-(pga1)-glutathione	206.719
5	ZINC000004654845	3-Hexaprenyl-4-hydroxybenzoate	205.355
6	ZINC000085541163	(+-)-Grossamide	204.523
7	ZINC000008552069	Thf-L-glutamate	202.145
8	ZINC000013513540	1,14-Bis(dihydrocaffeoyl)spermine	201.87
9	ZINC000004228293	5-Formiminotetrahydrofolate	201.743
10	ZINC000009212428	Leucal	200.227
11	ZINC000014952116	Enkephalin	197.698
12	ZINC000014712793	Kukoamine B	196.511
13	ZINC000011616636	Hibon	196.025
14	ZINC000008220036	2-Hexaprenyl-3-methyl-6-methoxy-1,4 benzoquinone	195.582
15	ZINC000004096653	Dhhpba	195.52
16	ZINC000012494317	Isodesmosin	192.893
17	ZINC000014951658	Endomorphin 1	192.844
18	ZINC000085826835	(+-)-Grossamide	192.709
19	ZINC000042805482	Grossamide	192.559
20	ZINC000004097774	Lithospermic acid	192.238

**Table 2:** ADME (adsorption, distribution, metabolism, and excretion) properties of compounds.

Number	ZINC ID	Solubility level^a^	BBB level^b^	CYP2D6 prediction^c^	Hepatotoxicity^d^	Absorption level^e^	PPB prediction^f^
1	ZINC000062238222	3	4	0	1	3	0
2	ZINC000085544839	3	4	0	1	3	0
3	ZINC000095620524	4	4	0	1	3	0
4	ZINC000004099069	3	4	0	0	3	0
5	ZINC000004654845	1	4	1	0	3	1
6	ZINC000085541163	2	4	0	0	2	0
7	ZINC000008552069	4	4	0	1	3	0
8	ZINC000013513540	4	4	0	1	3	0
9	ZINC000004228293	4	4	0	1	3	0
10	ZINC000009212428	4	4	0	1	3	0
11	ZINC000014952116	4	4	0	0	3	0
12	ZINC000014712793	4	4	0	0	3	0
13	ZINC000011616636	2	4	0	0	3	0
14	ZINC000008220036	0	4	1	0	3	1
15	ZINC000004096653	1	4	0	0	3	1
16	ZINC000012494317	1	4	1	0	3	0
17	ZINC000014951658	3	4	0	0	3	0
18	ZINC000085826835	2	4	0	0	2	0
19	ZINC000042805482	2	4	0	0	2	0
20	ZINC000004097774	2	4	0	0	3	0
21	6U3X	/	/	0	1	/	0

^a^Aqueous solubility level: 0 (extremely low); 1 (very low, but possible); 2 (low); and 3 (good); ^b^blood-brain barrier level: 0 (very high penetrant); 1 (high); 2 (medium); 3 (low); and 4 (undefined); ^c^cytochrome P450 2D6 level: 0 (noninhibitor) and 1 (inhibitor); ^d^hepatotoxicity: 0 (nontoxic) and 1 (toxic); ^e^human intestinal absorption level: 0 (good); 1 (moderate); 2 (poor); and 3 (very poor); ^f^plasma protein binding: 0 (binding is <90%); 1 (binding is >90%); and 2 (binding is >95%).

**Table 3:** Toxicities of compounds.

Number	ZINC ID	Rat NTP^a^		Mouse NTP^a^		AMES^b^	DTP^c^
		Male	Female	Male	Female		
1	ZINC000062238222	0.969	0.000	0.000	0.000	0.989	1.000
2	ZINC000085544839	0.964	0.000	0.080	0.000	0.999	1.000
3	ZINC000095620524	0.999	1.000	0.000	1.000	0.000	1.000
4	ZINC000004099069	0.000	0.000	0.000	0.000	0.002	0.864
5	ZINC000004654845	0.000	1.000	1.000	0.000	1.000	1.000
6	ZINC000085541163	0.998	1.000	1.000	0.186	0.000	1.000
7	ZINC000008552069	0.997	0.000	0.000	0.015	1.000	1.000
8	ZINC000013513540	0.840	0.020	0.000	0.139	0.000	1.000
9	ZINC000004228293	1.000	0.000	0.000	0.165	0.000	1.000
10	ZINC000009212428	1.000	0.000	0.000	1.000	0.356	1.000
11	ZINC000014952116	0.000	0.000	0.000	0.001	0.000	0.928
12	ZINC000014712793	0.632	1.000	0.000	0.640	0.000	1.000
13	ZINC000011616636	1.000	1.000	1.000	0.000	1.000	1.000
14	ZINC000008220036	0.000	1.000	1.000	0.000	0.064	0.000
15	ZINC000004096653	0.000	1.000	1.000	0.000	1.000	1.000
16	ZINC000012494317	0.000	0.000	0.000	0.222	0.000	1.000
17	ZINC000014951658	0.000	1.000	0.000	1.000	0.000	1.000
18	ZINC000085826835	0.998	1.000	1.000	0.186	0.000	1.000
19	ZINC000042805482	0.998	1.000	1.000	0.186	0.000	1.000
20	ZINC000004097774	0.053	0.987	0.916	0.000	0.002	1.000
21	6U3X	1.000	0.139	0.012	0.002	1.000	1.000

^a^0 (noncarcinogen) and 1 (carcinogen); ^b^0 (nonmutagen) and 1 (mutagen); ^c^0 (nontoxic) and 1 (toxic).

**Table 4:** CDOCKER interaction energy of compounds with PCSK9.

ZINC ID	CDOCKER interaction energy (Kcal/mol)
ZINC000004099069	-87.8609
ZINC000014952116	-65.9632

**Table 5:** Hydrogen bond interaction parameters for potential compounds and PCSK9.

Receptor	ZINC ID	Donor atom	Receptor atom	Distances (Å)
PCSK9	ZINC000004099069	B:ARG476:HH11	ZINC000012494317:N22	2.52581
		ZINC000012494317:H38	B:ARG357:O	2.05695
		ZINC000012494317:H38	B:VAL359:O	2.3965
		ZINC000012494317:H39	B:VAL359:O	2.0501
		ZINC000012494317:H57	B:CYS477:O	2.32261
		ZINC000012494317:H60	B:GLU332:OE2	2.18568
		ZINC000012494317:H71	B:ILE474:O	2.08114
		ZINC000012494317:H84	B:PRO331:O	2.25439
		ZINC000012494317:H86	B:PRO331:O	2.22788
		ZINC000012494317:H86	B:VAL333:O	2.64061
		ZINC000012494317:H87	B:VAL333:O	2.1696
		ZINC000012494317:H87	B:ASP360:OD2	2.22538
		ZINC000012494317:H89	B:LEU436:O	1.85936
		ZINC000012494317:H90	B:LEU436:O	2.0148
		B:PRO331:HA	ZINC000012494317:N31	2.49123
		B:TRP461:HD1	ZINC000012494317:O36	2.22741
		B:ALA478:HA	ZINC000012494317:O16	2.67059
		ZINC000012494317:H48	B:ARG357:O	2.56198
		ZINC000012494317:H59	B:PRO331:O	2.82566
		ZINC000012494317:H85	B:CYS358:O	2.49137
		ZINC000012494317:H85	B:ASP360:OD2	2.83867
		ZINC000012494317:H88	B:THR459:O	2.44502
	ZINC000014952116	B:ARG357:HH12	ZINC000014952116:O8	2.89478
		B:ARG357:HH22	ZINC000014952116:O40	1.86693
		B:ARG458:HH11	ZINC000014952116:O35	2.07486
		B:ARG458:HH12	ZINC000014952116:O23	2.05105
		B:TRP461:HN	ZINC000014952116:O27	2.7019
		ZINC000014952116:H60	ZINC000014952116:O23	1.92783
		ZINC000014952116:H63	B:ARG357:O	1.9849
		ZINC000014952116:H68	B:LEU436:O	1.85726
		ZINC000014952116:H74	B:ASP360:OD1	2.1021
		B:PRO331:HA	ZINC000014952116:O19	2.69646
		B:VAL460:HA	ZINC000014952116:O27	2.85792
		B:TRP461:HD1	ZINC000014952116:O27	2.13275
		ZINC000014952116:H61	B:ASP360:OD1	2.55504
		ZINC000014952116:H62	B:CYS358:O	2.45617
		ZINC000014952116:H62	B:ASP360:OD2	3.01446
		ZINC000014952116:H65	B:ARG357:O	2.40176
		ZINC000014952116:H77	ZINC000014952116:O8	2.41561
		B:ARG458:HH22	ZINC000014952116	2.79122

**Table 6:** Hydrophobic interaction parameters for compounds and PCSK9 residues.

Receptor	ZINC ID	Donor atom	Receptor atom	Distances (Å)
PCSK9	ZINC000004099069	B:VAL460	ZINC000012494317	4.51394
	ZINC000014952116	ZINC000014952116	B:ALA478	4.83821
		ZINC000014952116	B:ILE416	4.45717
		ZINC000014952116	B:PRO438	5.38467
		ZINC000014952116	B:ARG458	4.74229

## Data Availability

The data used and analyzed in this study are available upon reasonable request and can be found in the article/Supplementary Material.

## References

[1] Kobiyama K., Ley K. (2018). Atherosclerosis. *Circulation Research*.

[2] Macchi C., Ferri N., Sirtori C. R., Corsini A., Banach M., Ruscica M. (2021). Proprotein convertase subtilisin/kexin type 9: a view beyond the canonical cholesterol-lowering impact. *The American Journal of Pathology*.

[3] Myler R. K., Ryan C., Dunlap R. (1995). Dyslipoproteinemias in atherosclerosis, thrombosis and restenosis after coronary angioplasty. *The Journal of Invasive Cardiology*.

[4] Mourikis P., Zako S., Dannenberg L. (2020). Lipid lowering therapy in cardiovascular disease: from myth to molecular reality. *Pharmacology & Therapeutics*.

[5] Ference B. A., Robinson J. G., Brook R. D. (2016). Variation inPCSK9andHMGCRand risk of cardiovascular disease and diabetes. *The New England Journal of Medicine*.

[6] Heron M. (2019). Deaths: leading causes for 2017. *National Vital Statistics Reports*.

[7] Genser B., März W. (2006). Low density lipoprotein cholesterol, statins and cardiovascular events: a meta-analysis. *Clinical Research in Cardiology*.

[8] Seidah N. G., Prat A. (2022). The multifaceted biology of PCSK9. *Endocrine Reviews*.

[9] Brown M. S., Goldstein J. L. (1986). A receptor-mediated pathway for cholesterol homeostasis. *Science*.

[10] Cunningham D., Danley D. E., Geoghegan K. F. (2007). Structural and biophysical studies of PCSK9 and its mutants linked to familial hypercholesterolemia. *Nature Structural & Molecular Biology*.

[11] Maxwell K. N., Breslow J. L. (2004). Adenoviral-mediated expression of Pcsk9 in mice results in a low-density lipoprotein receptor knockout phenotype. *Proceedings of the National Academy of Sciences of the United States of America*.

[12] Poirier S., Mayer G., Benjannet S. (2008). The proprotein convertase PCSK9 induces the degradation of low density lipoprotein receptor (LDLR) and its closest family members VLDLR and ApoER2. *The Journal of Biological Chemistry*.

[13] Klein-Szanto A. J. P., Bassi D. E. (2019). Keep recycling going: new approaches to reduce LDL-C. *Biochemical Pharmacology*.

[14] Santos R. D., Ruzza A., Hovingh G. K. (2020). Evolocumab in pediatric heterozygous familial hypercholesterolemia. *The New England Journal of Medicine*.

[15] Hovingh G. K., Lepor N. E., Kallend D., Stoekenbroek R. M., Wijngaard P. L. J., Raal F. J. (2020). Inclisiran durably lowers low-density lipoprotein cholesterol and proprotein convertase subtilisin/kexin type 9 expression in homozygous familial hypercholesterolemia. *Circulation*.

[16] Ding Q., Strong A., Patel K. M. (2014). Permanent alteration of PCSK9 with in vivo CRISPR-Cas9 genome editing. *Circulation Research*.

[17] Newman D. J. (2016). Developing natural product drugs: supply problems and how they have been overcome. *Pharmacology & Therapeutics*.

[18] Yang L., Li W., Zhao Y. (2019). Computational study of novel natural inhibitors targeting O^6^-methylguanine-DNA methyltransferase. *World Neurosurgery*.

[19] Bang S., Chae H. S., Lee C. (2017). New aromatic compounds from the fruiting body of Sparassis crispa (Wulf.) and their inhibitory activities on proprotein convertase subtilisin/kexin type 9 mRNA expression. *Journal of Agricultural and Food Chemistry*.

[20] Ray K. K., Landmesser U., Leiter L. A. (2017). Inclisiran in patients at high cardiovascular risk with elevated LDL cholesterol. *The New England Journal of Medicine*.

[21] Fitzgerald K., White S., Borodovsky A. (2017). A highly durable RNAi therapeutic inhibitor of PCSK9. *The New England Journal of Medicine*.

[22] Stucchi M., Grazioso G., Lammi C. (2016). Disrupting the PCSK9/LDLR protein-protein interaction by an imidazole-based minimalist peptidomimetic. *Organic & Biomolecular Chemistry*.

[23] Li L., Shen C., Huang Y. X. (2018). A new strategy for rapidly screening natural inhibitors targeting the PCSK9/LDLR interaction in vitro. *Molecules*.

[24] Ahmad P., Alvi S. S., Iqbal D., Khan M. S. (2020). Insights into pharmacological mechanisms of polydatin in targeting risk factors-mediated atherosclerosis. *Life Sciences*.

[25] Min D. K., Lee H. S., Lee N. (2015). In silico screening of chemical libraries to develop inhibitors that hamper the interaction of PCSK9 with the LDL receptor. *Yonsei Medical Journal*.

[26] Taechalertpaisarn J., Zhao B., Liang X., Burgess K. (2018). Small molecule inhibitors of the PCSK9.LDLR interaction. *Journal of the American Chemical Society*.

[27] Petrilli W. L., Adam G. C., Erdmann R. S. (2020). From screening to targeted degradation: strategies for the discovery and optimization of small molecule ligands for PCSK9. *Cell Chemical Biology*.

[28] Sharifi-Rad J., Sureda A., Tenore G. C. (2017). Biological activities of essential oils: from plant chemoecology to traditional healing systems. *Molecules*.

[29] Kesarwani K., Gupta R., Mukerjee A. (2013). Bioavailability enhancers of herbal origin: an overview. *Asian Pacific Journal of Tropical Biomedicine*.

[30] Yang B., Mao J., Gao B., Lu X. (2019). Computer-assisted drug virtual screening based on the natural product databases. *Current Pharmaceutical Biotechnology*.

